# Exploring the Association of Polypharmacy and Depressive Symptoms with Sarcopenia in Older Adults

**DOI:** 10.3390/jcm15145633

**Published:** 2026-07-17

**Authors:** Bader A. Alqahtani, Aqeel M. Alenazi

**Affiliations:** Department of Health and Rehabilitation Sciences, College of Applied Medical Sciences, Prince Sattam Bin Abdulaziz University, Al-Kharj 11942, Saudi Arabia

**Keywords:** sarcopenia, medications, mental health

## Abstract

**Objectives**: This study aimed to investigate the prevalence of and risk factors for sarcopenia in community-dwelling older adults. **Methods**: This cross-sectional study was conducted in Riyadh between February and May 2024. Sarcopenia was diagnosed using the Asian Working Group on Sarcopenia (AWGS) criteria, which include muscle mass, grip strength, and gait speed. Polypharmacy was defined as taking 5 medications or more. Depressive symptoms were examined using the Arabic version of the Geriatric Depression Scale-15 with a cutoff score of >5. Multivariable logistic regression analyses were used to assess the possible risk factors for sarcopenia. **Results**: A total of 182 participants were enrolled and analyzed in the current study. Approximately 11% (*n* = 20) of participants were identified as having sarcopenia based on the AWGS criteria. Individuals with sarcopenia were older, with a mean age of 68.6 years, and a higher percentage were female (75.0% vs. 41.4% in the non-sarcopenia group; *p* = 0.004). Compared with the non-sarcopenia group, the sarcopenia group showed a higher prevalence of polypharmacy (≥5 medications) (55% vs. 16%; *p* < 0.001) and depressive symptoms (55% vs. 13.6%; *p* < 0.001). **Conclusions**: This study identified an increased prevalence of sarcopenia among the geriatric population in the Kingdom of Saudi Arabia using the AWGS criteria. The associated risk factors for sarcopenia included older age, polypharmacy, and depressive symptoms. These associations underscore the complex systemic interplay between physical health, mental well-being, and therapeutic management in geriatric care. Routine assessment for sarcopenia should be implemented into primary healthcare protocols for the older population, along with medication review and depressive symptom screening.

## 1. Introduction

Sarcopenia is an age-related progressive condition characterized by a general decrease in skeletal muscle mass, strength, and physical performance [1]. Rosenberg coined the term “sarcopenia” to refer to a decrease in muscular mass in older adults [2]. Over the last few decades, researchers have shifted their focus from concentrating solely on muscle mass to a broader definition of sarcopenia, which includes muscle strength and physical function. The European Working Group on Sarcopenia in Older People (EWGSOP) defines sarcopenia as a condition that involves a gradual and general loss of skeletal muscle mass and strength, with a risk of physical impairment, mortality, and low quality of life [3]. Sarcopenia was first recognized as a disease in 2016 [4].

Sarcopenia has been associated with poor outcomes, including physical disability, comorbidities, risk of falls, injuries, hospital admission, and death rates, creating a greater burden on the healthcare system [1,5,6,7,8]. Previous studies have linked sarcopenia to cognitive decline, functional limitations, polypharmacy, and depressive symptoms [9,10]. The healthcare system is significantly affected by sarcopenia in economic terms. Individuals with sarcopenia incurred healthcare expenditures five times higher than those of hospitalized older individuals without sarcopenia [11]. Additionally, lowering sarcopenia by 10% may result in yearly savings of more than one billion dollars [12].

There is a discrepancy in the literature regarding the global prevalence of sarcopenia, which can be attributed to different diagnostic criteria and the heterogeneity of the studied populations worldwide. According to a recent review, the prevalence of sarcopenia in older adults generally falls between 10% and 16%, based on the specific diagnostic criteria utilized and the group investigated [9]. Another systematic review and meta-analysis reported an overall prevalence of sarcopenia of 27% based on muscle mass in the general population [13]. However, previous studies conducted in Saudi Arabia were limited either by using SARC-F as a screening tool rather than a diagnostic criterion or by restricting the sample to pre-frail participants or including only women. These limitations may limit the generalizability of the results to the broader Saudi older adult population. A previous study used the SARC-F questionnaire to estimate sarcopenia prevalence in this cohort; however, this method provides inconsistent results, has low sensitivity [14], and cannot serve as a standalone diagnostic tool. Another study included only women [15,16]. This distinction is important because the prevalence of sarcopenia varies substantially according to the diagnostic criteria. Furthermore, polypharmacy has been linked to sarcopenia in previous research, and depressive symptoms have also been associated with sarcopenia, supporting their inclusion as potential risk factors [10,17,18]. Therefore, the current study aimed to accurately assess the prevalence of sarcopenia in a geriatric population living in the Kingdom of Saudi Arabia using well-established criteria. In addition, with current demographic trends indicating gradual aging of the Saudi population, the prevalence of sarcopenia is likely to increase, posing a significant public health problem. This highlights the importance of conducting rigorous, detailed, and up-to-date evaluations to implement effective preventive and intervention strategies. Therefore, this study aimed to assess the prevalence of sarcopenia and its associated risk factors, including polypharmacy and depressive symptoms, in older adults living in Saudi Arabia.

## 2. Methods

This cross-sectional study was conducted primarily in the Riyadh area of the Kingdom of Saudi Arabia using a convenience sampling approach. The study was conducted between February and May 2024. We recruited participants primarily through local residential neighborhoods, public advertising, and media. The inclusion criteria included being 60 years of age or older, being fluent in Arabic, and being able to ambulate independently. Participants were excluded if they had mild cognitive impairment, as the study outcomes, including medication use and depressive symptoms, required sufficient cognitive ability for accurate recall and reporting. Cognitive function was assessed using the Montreal Cognitive Assessment (MoCA) [19]. Participants with acute diseases that might influence their responses were also excluded. Written informed consent was obtained from each participant, in accordance with the Declaration of Helsinki. Ethical approval (NO. RHPT/24/3, 5 January 2024) was issued by the Ethics Committee of Prince Sattam bin Abdulaziz University.

### 2.1. Sample Size Estimation

An a priori power calculation was conducted using G*Power 3.1 to determine the required sample size for sarcopenia prevalence. The sample size was estimated based on previous evidence of the prevalence of sarcopenia [13]. The estimated overall prevalence of sarcopenia based on muscle mass was 27%. Therefore, this criterion was included in the power calculations. We used a chi-square goodness-of-fit test with an effect size of 0.27 (medium effect), a significance level (α) of 0.05, a desired power (1 − β) of 0.80, and 5 degrees of freedom. Finally, the analysis yielded the minimum required sample size of 176 participants for the current study. A total of 182 participants were included in the study.

### 2.2. Outcome Measures

#### Demographics

Data on age, sex, marital status, medications, chronic conditions, and height and weight, which were used to calculate body mass index (BMI), were collected. The medical history of chronic conditions was obtained through participant self-report during the assessment session. Participants verified the number of medications they took. Medication history was also collected via self-report to determine the total number of medications currently being used for further analysis. Polypharmacy was defined as the use of five or more medications by participants in the current sample [20].

### 2.3. Definitions of Sarcopenia

We used the Asian Working Group on Sarcopenia (AWGS) diagnostic criteria. Although developed for East Asian groups, the AWGS cutoffs have been applied in Saudi and Middle Eastern studies [16,21]. Validation of the AWGS criteria in Saudi Arabia remains limited; however, these criteria enable meaningful regional and international comparisons. The AWGS include low grip strength (<27 kg for males and <18 kg for females), low muscle mass (<7 kg/m^2^ for males and <5.7 kg/m^2^ for females), and gait speed of ≤1 m/s [22]. Participants with sarcopenia was defined when participants have low muscle mass with low muscle strength or poor physical performance, i.e., gait speed.

### 2.4. Sarcopenia-Related Measures

#### 2.4.1. Muscle Mass

Muscle mass is a recognized diagnostic criterion in the AWGS. To measure muscle mass, body composition was assessed using bioelectrical impedance analysis (BIA) (BIACORPUS RX 4004M, MEDICAL HealthCare GmbH, Karlsruhe, Germany) in the current study. BIA was selected based on its practical use for estimating appendicular skeletal muscle mass in epidemiological and clinical settings where DXA is not feasible [23]. A validated equation developed by Sergie et al. was utilized to estimate appendicular skeletal muscle mass (ASMM): ASMM (kg) = −3.964 + (0.227 × (height^2^/Resistance)) + (0.095 × weight) + (1.384 × sex (with male = 1; female = 0)) + (0.064 × Reactance) [24]. This equation was used because it was validated against DXA and demonstrated strong predictive performance.

#### 2.4.2. Muscle Strength

Handgrip strength was measured using a hand dynamometer (JAMAR PLUS+ Sammons Preston, Bolingbrook, IL, USA) and reported in kilograms. The research assistants who measured handgrip strength were two physical therapists trained in data collection procedures using a standard protocol. The dominant hand was measured three times, and the average value was used for analysis.

#### 2.4.3. Gait Speed

A 4 m track was used to measure gait speed. Gait speed is a simple measure and functional mobility test with good reliability and validity in older adults. Each participant was instructed to walk at a normal pace and allowed to use an assistive device if needed during the test, with 1 m acceleration and 1 m deceleration zones. The research assistant used a stopwatch to time each trial. Gait speed (m/s) was calculated by dividing the distance by the time required to complete the test.

### 2.5. Geriatric Depression Scale-15 (GDS-15; Arabic Version)

The GDS-15 short version was developed to screen for depressive symptoms in older adults. The GDS-15 consists of 15 questions regarding the participants’ mood. All the answers pertain to the participants’ experiences during the previous week. All answers have “yes” or “no” response options, with 1 point assigned to either “yes” or “no,” depending on the question [25]. A score > 5 was used as the cutoff score to suggest depressive symptoms. In the current study, the Arabic-GDS-15 was used to measure depressive symptoms. This scale has been shown to be valid and reliable, with high internal consistency (ICC = 0.89) [26].

### 2.6. Statistical Analysis

For continuous data, means and standard deviations were used, whereas for categorical data, percentages and frequencies were reported. The normality of the data was assessed using the Kolmogorov–Smirnov test. Multivariable logistic regression analyses were used to examine the risk factors associated with sarcopenia in this sample, with associated odds ratios (ORs) and 95% confidence intervals (95% CIs). The goodness of fit of the model was assessed by the Hosmer–Lemeshow test, which indicated an adequate model fit (χ^2^ = 5.14, *p* = 0.742). A *p*-value of less than 0.05 was set as the significance level. All analyses used Stata version 15.1 (StataCorp, College Station, TX, USA) for data processing.

## 3. Results

A total of 182 participants were enrolled and analyzed in this study. Approximately 11% (*n* = 20) were categorized as having sarcopenia based on the AWGS criteria. There were significant differences in several baseline characteristics between the sarcopenia and non-sarcopenia groups. Participants with sarcopenia were older, with a mean age of 68.6 years, and a higher percentage were female (75.0% vs. 41.4% in the non-sarcopenia group; *p* = 0.004). Additionally, the sarcopenia group had a higher prevalence of polypharmacy (≥5 medications) (55% vs. 16%; *p* < 0.001) and depressive symptoms (55% vs. 13.6%; *p* < 0.001) than the non-sarcopenia group. This group also showed significantly lower physical performance, indicated by lower grip strength (15.6 kg vs. 27.5 kg) and walking speed (0.70 m/s vs. 1.03 m/s). There were no notable differences in body mass index (BMI) or MoCA cognitive scores between the two groups (Table 1).

**Table 1:** Socio-demographic characteristics of participants in the study.

Variable	Total (*n* = 182)	AWGS Criteria		*p* Value
		Non-Sarcopenia (*n* = 162)	Sarcopenia (*n* = 20)	
Age (y)	65.6 ± 5.2	65.2 ± 5.2	68.6 ± 4.7	0.006
Female, *n* (%)	82 (45.1)	67 (41.4)	15 (75.0)	0.004
Polypharmacy, *n* (%)				<0.001
<5 Number of medications	145 (79.7)	136 (84.0)	9 (45.0)	
≥5 Number of medications	37 (20.3)	26 (16.0)	11 (55.0)	
MoCA, mean (SD)	27.9 (1.3)	27.9 (1.3)	27.9 (1.3)	0.7713
Grip strength, mean (SD)	26.2 ± 8.8	27.5 ± 8.2	15.6 ± 5.9	<0.001
Gait speed (m/s), mean score (SD)	0.99 ± 0.31	1.03 ± 0.30	0.70 ± 0.18	<0.001
Depressive symptoms *n* (%)				<0.001
No	149 (81.9)	140 (86.4)	9 (45.0)	
Yes	33 (18.1)	22 (13.6)	11 (55.0)	
BMI (kg/m^2^), mean (SD)	29.8 ± 5.4	29.6 ± 5.3	31.4 ± 5.8	0.157
MOCA: Montreal Cognitive Assessment; BMI: Body Mass Index				

The factors associated with sarcopenia are shown in Table 2. Age, use of five or more medications, and depressive symptoms were significantly associated with sarcopenia (OR  =  1.12, 95% CI  =  1.0–1.2; OR  =  3.4, 95% CI  =  1.1–10.5; OR  =  4.4, 95% CI  =  1.4–13.3, respectively). There was a higher proportion of male participants (54.9%) than female participants (45.1%). This distribution reflects natural recruitment patterns in community settings in Riyadh. Although a higher proportion of females was observed in the sarcopenia group in the unadjusted analysis, sex was not significantly associated with sarcopenia in the multivariable logistic regression model (adjusted OR 6.2, 95% CI 0.91–10.6). Therefore, this imbalance is unlikely to have substantially influenced the findings of the main study.

**Table 2:** Multiple regression analysis between factors associated with sarcopenia.

Variables	Adjusted OR (95% CI)	*p*
Age	1.12 (1.0–1.2)	0.016
Sex		
Female	3.1 (0.92–10.6)	0.068
Polypharmacy ≥ 5 number of medications	3.4 (1.1–10.5)	0.028
Depressive symptoms		
Yes	4.4 (1.4–13.3)	0.009

OR: Odds Ratio.

## 4. Discussion

This study established the prevalence of sarcopenia among community-dwelling older adults in Saudi Arabia and its associated risk factors. According to the AWGS criteria, the prevalence of sarcopenia in this population was 11%. The risk factors associated with sarcopenia in the present study were older age, polypharmacy, and depressive symptoms. However, sex was not significantly associated with sarcopenia in the adjusted analysis. This study was the first in the region to include both men and women from the community in Saudi Arabia.

The prevalence of sarcopenia in the current study (11%) was consistent with previous international reports. A previous meta-analysis examined the prevalence of sarcopenia in the general population [27]. This study reported a pooled prevalence of sarcopenia of 10% across 35 studies, including a total of 58,404 participants [27]. The pooled prevalence (10%) was consistent with the results of the present study (11%). However, the current study was in contrast to other meta-analyses, including 70 studies that reported a range of sarcopenia prevalence between 5.2% and 62.7% in the community [28]. This variability in the prevalence of sarcopenia may be attributed to the lack of a pooled prevalence and further analysis in this study. Other studies from different countries have reported different prevalences of sarcopenia, including China (6.4% in men and 11.5% in women) [29], the United States (36.5%) [30], Vietnam (32%) [31], Korea (27.5%) [32], and Turkey (5.2%) [33], compared with the current study (11%). These differences could be attributed to the different diagnostic criteria and populations with variations in lifestyle, physical activity levels, and demographic factors. Few studies have examined the prevalence of sarcopenia in Saudi Arabia [15,34]. These studies reported a higher prevalence of sarcopenia, ranging from 20% to 65%, using different methods and definitions, which was higher than that reported in the current study (11%) [14]. The differences between the current study and previous studies in Saudi Arabia included the use of participants from hospitals with specific conditions, such as pre-frailty or only females, and the application of different diagnostic criteria, including the SARC-F. The current study included both male and female participants from the community using the AWGS criteria.

The current study found that older age was associated with sarcopenia. These results were consistent with previous evidence that found an association between older age and sarcopenia in different countries, including Vietnam [31], Korea [32], Thailand [35], and Turkey [33]. However, the significant association with older age in the current study was inconsistent with that of a previous study in Saudi Arabia [36]. This might be attributed to different sampling methods, as this study recruited patients visiting a hospital with a high prevalence of disability and the need for home care assistance, whereas the current study recruited participants from the community who were able to perform functional tasks. Older age may be linked to sarcopenia due to a reduction in growth hormone factors and an increase in inflammatory markers, resulting in muscle mass loss with advanced age [37,38].

Polypharmacy has been linked to sarcopenia due to the impact of medications on overall health, medication interactions, and side effects. Previous studies identified a relationship between polypharmacy and sarcopenia in community-dwelling older adults [10,17,39,40]. In the current study, the prevalence of polypharmacy among participants with sarcopenia was 55%. The results of the current study were consistent with those of previous studies and this study was the first to report an association between polypharmacy and sarcopenia in older adults in Saudi Arabia. Polypharmacy may include specific drugs that have a negative effect on muscles, such as corticosteroids [41,42,43]. Another factor associated with polypharmacy and sarcopenia is the change in body weight and composition, which may affect drug metabolism and absorption [44]. Certain frequently prescribed medications for older adults have distinct risks of physiological decline. Medications, including glucocorticoids and antidepressants, may have a negative impact on muscles, leading to muscle tissue damage. Conversely, other common classes of drugs, such as non-steroidal anti-inflammatory drugs (NSAIDs) and beta-blockers, are known to cause adverse systemic metabolic changes, including impaired mitochondrial function, decreased blood perfusion, and subsequent disruptions in hormonal and electrolyte balance [17]. Further research is needed to examine the specific classes or clusters associated with sarcopenia in older adults.

In the current study, depressive symptoms were associated with sarcopenia, which is consistent with previous evidence [45]. In the current study, the prevalence of depressive symptoms was 55% among the participants with sarcopenia. This high prevalence may indicate that both sarcopenia and depressive symptoms share common risk factors. Common risk factors included low levels of physical activity and exercise, the upregulation of inflammatory markers, and hormonal disorders [46]. The association between sarcopenia and depressive symptoms is driven by several overlapping pathways, including the function of neurotrophic factors, systemic oxidative stress, chronic inflammation, and the regulation of behavioral factors related to daily living [47,48,49]. Future research should examine the causal relationship between depressive symptoms and sarcopenia using longitudinal studies.

Although this study included participants from the community and used standard diagnostic criteria for sarcopenia, including the AWGS criteria, it has some limitations that need to be considered. The small sample size may limit the generalizability of the findings. Additionally, the small sample size in the sarcopenia group limited the number of variables included in the multivariable logistic regression model to prevent overfitting. The cross-sectional design limited causal inference in the current study. Therefore, longitudinal studies are needed to determine the causal relationship between polypharmacy, depressive symptoms, and sarcopenia. Furthermore, the lack of gold-standard measures, such as dual-energy X-ray absorptiometry (DEXA), computed tomography, and magnetic resonance imaging for measuring muscle mass, is another limitation. The use of the Sergi equation in the current study was based on previous validation using DEXA. However, further validation is needed in future research as the equation may differ across different ethnic groups. Polypharmacy was obtained via self-report, which may have affected the results and led to recall bias. Future studies should use medications dispensed from the pharmacy records and other verification methods. Comorbidity was obtained via self-report, which may have introduced an underestimation or overestimation of chronic conditions. Finally, several important confounding factors known to be associated with sarcopenia were not assessed in this study, including physical activity level, nutritional status (e.g., protein intake), smoking status, and comorbidity burden. The absence of these variables may have influenced the associations observed. Further research is needed, including the assessment of additional confounding variables and the use of gold-standard measures for diagnosing sarcopenia.

## 5. Conclusions

The current study identified an increased prevalence of sarcopenia (11%) among older individuals in the Kingdom of Saudi Arabia according to the AWGS criteria. The associated risk factors for sarcopenia included older age, polypharmacy, and depressive symptoms in this population. These associations underscore the complex interplay between physical health, mental well-being, and therapeutic management in geriatric care. Future studies should examine whether routine screening for sarcopenia should be integrated into primary healthcare protocols for older adults, along with medication review and depressive symptom screening.

## Data Availability

The datasets used and/or analyzed in the current study are available from the corresponding author upon reasonable request.
